# Genome-Wide Association Study of Grain Number in Common Wheat From Shanxi Under Different Water Regimes

**DOI:** 10.3389/fpls.2021.806295

**Published:** 2022-01-26

**Authors:** Xingwei Zheng, Ling Qiao, Ye Liu, Naicui Wei, Jiajia Zhao, Bangbang Wu, Bin Yang, Juanling Wang, Jun Zheng

**Affiliations:** ^1^State Key Laboratory of Sustainable Dryland Agriculture, Institute of Wheat Research, Shanxi Agricultural University, Linfen, China; ^2^College of Life Science, Shanxi University, Taiyuan, China

**Keywords:** water regime, Shanxi wheat, GNS, GWAS, DTC

## Abstract

Water availability is a crucial environmental factor on grain number in wheat, which is one of the important yield-related traits. In this study, a diverse panel of 282 wheat accessions were phenotyped for grain number per spike (GNS), spikelet number (SN), basal sterile spikelet number (BSSN), and apical sterile spikelet number (ASSN) under different water regimes across two growing seasons. Correlation analysis showed that GNS is significantly correlated with both SN and BSSN under two water regimes. A total of 9,793 single nucleotide polymorphism (SNP) markers from the 15 K wheat array were employed for genome-wide association study (GWAS). A total of 77 significant marker-trait associations (MTAs) for investigated traits as well as 8 MTAs for drought tolerance coefficient (DTC) were identified using the mixed linear model. Favored alleles for breeding were inferred according to their estimated effects on GNS, based on the mean difference of varieties. Frequency changes in favored alleles associated with GNS in modern varieties indicate there is still considerable genetic potential for their use as markers for genome selection of GNS in wheat breeding.

## Introduction

Wheat (*Triticum aestivum* L.) is one of the most important crops globally, mainly grown in semiarid and arid regions of the world (Khan et al., 2019). To keep pace with the expanding global population, wheat yield is projected to increase 60% by 2050 (Ray et al., 2013; Cao et al., 2020), whereas wheat production will inevitably be affected by abiotic stresses, such as drought stress. As reported, significant wheat yield losses of 40% in less-developed irrigated growing areas occurred (Joshi et al., 2007). Therefore, it is imperative to scale up wheat yield under water deficit conditions, thus ensuring food security.

Wheat yield is determined by three factors, namely, spike number per unit area, grain number per spike (GNS), and thousand grain weight (TGW), which are important grain yield components (Shi et al., 2017). Recent studies have suggested that wheat grain yield is affected more by variation in GNS than by variation in grain size (Feng et al., 2018; Sakuma et al., 2019). GNS in wheat is determined by spikelet number and spikelet fertility, in addition to grain number per spikelet. Among these traits, spikelet number had higher heritability, whereas fertile spikelet number (SN) and grain number per spikelet were manipulatable with different environments. Molecular biology and genomics have become the key tools to understand the basis of GNS formation and deploying those genes for yield improvement (Cao et al., 2020). For example, the GNS-related genes in cereals, especially in rice, have a series of homologs that have been isolated from wheat by homology-based cloning, including *TaTEF* (Zheng et al., 2014), *FZP* (Dobrovolskaya et al., 2015), *TaTOC1* (Zhao et al., 2016), *TaSPL20*, and *TaSPL21* (Zhang et al., 2017). At the present, there are fewer genes related to the regulation of GNS in map-based cloning. For example, the floret fertility-regulated gene *GNI-A1* (Sakuma et al., 2019), triple-spikelet gene *WFZP* (Du et al., 2021), and aberrant panicle organization 1 gene *APO1* (Kuzay et al., 2019) were shown to be involved in regulating the formation of GNS in wheat. Accordingly, the identification of novel genetic loci controlling GNS is significant for broadening the genetic variation in molecularly designed wheat breeding.

Among the three main yield components in wheat, GNS is more affected by drought stress during the productive period than TGW (Fischer, 2008, 2011). Water deficiency directly affects both the vegetative and reproductive growth stages, ultimately reducing fertility parameters, grain yield components, and thus final yield (Ahmed et al., 2021; Jallouli et al., 2021). It seems likely that improvements in grain yield may derive from improvements in grain number, particularly under water stress conditions (Li et al., 2019; Sun et al., 2019; Zhang et al., 2020). Therefore, dissecting the genetic basis of grain number and its responses to water deficit is indispensable for the improvement of wheat.

To date, most of the reported quantitative trait loci (QTLs) controlling GNS and spikelet infertility were identified under high-yield potential conditions (Miura et al., 1992; Li et al., 2007; Ma et al., 2007; Jia et al., 2013; Zhai et al., 2016; Zhang H. et al., 2016; Cui et al., 2017; Deng et al., 2017). Relatively few studies have examined the consistency of QTLs under varying environmental stress conditions (Bilgrami et al., 2020). Therefore, it is essential to identify stable genetic loci for better agronomic performance, which can be selected for producing stable, high-yielding genotypes under diverse environments. The lack of major, stable genetic loci across multiple environments as well as the low-marker densities restricts the utilization efficiency for both marker-assisted selection and gene isolation (Ma et al., 2019). Despite this, most of the QTLs have been identified using biparental or multi-parental populations. The genetic variation of the population has been so far limited only to the genomes of the parents (Bilgrami et al., 2020). Based on the high-density single nucleotide polymorphisms (SNPs), genome-wide association study (GWAS) has been identified as an effective tool for discovering QTLs and genes associated with target traits in various crops such as wheat (Wang et al., 2014; Juliana et al., 2019). Shi et al. (2017) detected 62 significantly associated signals for kernel number per spike at 47 SNP loci on 19 chromosomes through GWAS. However, research to identify major stable loci of yield-related traits in wheat under water-stress conditions has been conducted using GWAS has been limited.

Shanxi Province in China is situated in a semiarid region, with an annual rainfall between 400 and 650 mm. Dryland occupies 70% of the wheat planting area. Shanxi has a long history of wheat planting and has always been famous for its drought resistance and stable yield varieties of wheat. Varieties such as Jinmai 33, Chang 6878, and Jinmai 47 were widely cultivated in dryland areas. The descendants of these excellent accessions are the main varieties currently spreading in China, thus making it a representative to study the genetic evolution of wheat GNS in semiarid areas and the effects of water regimes on GNS with Shanxi wheat varieties. In this study, 15 K SNP array markers were used to identify the population structure of the Shanxi wheat panel and genome-wide MTAs of wheat GNS, SN, BSSN, and ASSN under different water regimes. This association analysis provides useful information for marker-assisted selection in breeding wheat for increasing yield.

## Materials and Methods

### Plant Material

A total of 282 hexaploid wheat collections in Shanxi Province of China were used in this experiment (Supplementary Table 1). These genotypes differ by their origin and planting model, including 127 irrigated wheat cultivars, 115 dryland cultivars, and 40 landraces. These landrace samples are Chinese wheat mini core collection from Shanxi (Hao et al., 2011).

### Field Experiment

This study examined the results under two irrigation regimes at the experimental station of Linfen in Shanxi Province, China, located at 36°48′ N and 111°30′ E. The study was conducted over 2 consecutive years (2019–2020, 2020–2021). The monthly rainfall rates and average temperature during the two trial years are presented in Supplementary Figure 1. The rainfall amount during the months of October–May in 2019–2020 was 201 mm and in 2020–2021 was 111 mm. The regimes were conducted as irrigation: once (I1) at the overwintering stage and three times (I3) at overwintering, jointing, and booting stage. The wheat genotypes were assessed in controlled field conditions using a randomized complete block design with three replications. Each plot represented one experimental unit: a single-row plot of 1.5 m in length containing 21 seeds evenly distributed with 0.30 m spacing between rows. The field trial area was leveled before seeding to ensure that all plants would be under the same water level.

### Trait Phenotyping and Data Analysis

Ten representative primary tillers from the center of each row were collected to investigate the following traits: total SN per spike, the GNS, the basal sterile spikelet number (BSSN), and the apical sterile spikelet number (ASSN). After harvest, thousand grain weight (TGW) was measured. The drought tolerance coefficient (DTC) of GNS, SN, and TGW values was calculated using the formula I1/I3, while for BSSN and ASSN, it was calculated using I3/I1.

To eliminate environmental effects, the best linear unbiased prediction (BLUP) values across two repetitions were conducted using *R*. The *H*^2^ value was calculated using the formula *H*^2^ = *VG*/(*VG* + *VE*/*r*), where *VG* is the genotypic variance, *VE* is the environment variance, and *r* is the number of replications (Lin et al., 2020). Correlation analyses were performed using SPSS 20 (IBM SPSS Statistics; IBM Corp., Armonk, NY, United States).

### Single Nucleotide Polymorphism Genotyping

For the genotyping assay, approximately 1.0 g of a young leaf was collected from each wheat genotype before they reached the elongation stage. Genomic DNA was extracted using the cetyl trimethyl ammonium bromide (CTAB) method and stored at − 80°C until use. DNA dissolved in TE buffer was sent to MOL-BREEDING company (Shijiazhuang, China) for high-throughput genotyping using a set of GenoBaits Wheat 20995 (10111mSNP) panels. After filtering out markers with minimum allele frequency (MAF) <0.05 and markers with >10% missing data, as well as >20% heterozygosity (Jung et al., 2021), a total of 9,793 high-quality SNPs were included in the following population structure and GWAS analyses.

### Genome-Wide Association Analysis

TASSEL 5.0 was used to examine the associations between SNPs and phenotypic variations (Bradbury et al., 2007). SNPs-trait association was tested using the mixed linear model (MLM). A threshold *P*-value of < 0.001 or − log_10_(*P*-value) < 3 was used as the screening criterion (Guan et al., 2019). The linkage disequilibrium (LD) of each single SNP marker was extended on each chromosome. The extended region where the LD between nearby SNPs and the peak SNP decayed to *r*^2^ = 0.2 was defined as the local LD-based QTL interval (Zhang X. H. et al., 2016). Therefore, significant SNPs were selected with a physical distance ≤ LD-based interval and referred to as a conservative QTL.

## Results

### Phenotype Assessments

The phenotypes of 282 wheat accessions were characterized during two crop seasons (2019–2021) in I1 and I3 environments. Descriptive statistics data and frequency distribution of the genotypes for the investigated traits in I1 and I3 environments based on the average data over years are presented in Table 1 and Supplementary Figure 2, respectively. There was a significant genetic variation among accessions for all the traits in the two water conditions, the water treatments had highly significant effects on GNS and ASSN (*p* < 0.001; Table 1 and Supplementary Figure 2). In the I3 condition, averaged over 2 years, GNS of the wheat genotypes varied from 28.00 to 88.60, generated 16.75–25.80 SN, 0–5.80 BSSN, 0–3 ASSN, and weighted 17.00–57.75 g TGW. After water regimes were changed to I1, the genotypes varied in GNS from 22.50 to 68.00, generated 16.80–26.20 SN, 0–7.6 BSSN, 0–4.2 ASSN, and weighted 21.50–54.95 g TGW. Compared with the I1 treatment, under the I3 condition, the mean value for GNS was significantly increased by 26.63%, and SN and TGW were both slightly increased to 0.74 and 7.54%, respectively. The highest heritability was observed for TGW, GNS, and SN with values of *H*^2^ = 95.06, *H*^2^ = 87.76, and *H*^2^ = 82.40 (Table 1). Compared with I1, BSSN and ASSN were decreased by 7.53 and 21.15%, respectively.

**TABLE 1:** Analysis of variance in SN, GNS, BSSN, ASSN, and TGW of wheat under different irrigation conditions (I1 and I3) during the 2019–2020 and 2020–2021 growing seasons.

Traits	Water regimes	Descriptive statistics		Variance parameters			
		Mean	Range	G	G × E	E	H^2^ (%)
GNS	I1	41.12	22.50–68.00	18.16	49.15	32.68	87.76
(number)	I3	52.07	28.00–88.60	**	***	***	
SN	I1	21.50	16.80–26.20	42.94	56.08	0.98	82.40
(number)	I3	21.66	16.75–25.80	***	***		
BSSN	I1	2.00	0.00–7.60	33.19	49.25	17.57	68.18
(number)	I3	1.86	0.00–5.80	***	***		
ASSN	I1	0.63	0.00–4.20	8.455	72.28	19.274	26.46
(number)	I3	0.52	0.00–3.00		***	**	
TGW	I1	37.13	21.50–54.95	46.83	21.15	32.03	95.06
(g)	I3	39.93	17.00–57.75	***		**	

*Data were presented as the mean.*

** and *** represent significance level of P < 0.01 and P < 0.001.

*GNS, the grain number per spike; SN, total spikelet number per spike; BSSN, the basal sterile spikelet number; ASSN, the top sterile spikelet number; TGW, thousand-grain weight; I1, irrigation once at overwintering stage; I3, irrigation three times at overwintering, jointing, and booting stage.*

For the three different types, compared with I1, under the I3 irrigation regime, the highest increase in the mean value of GNS happened in irrigated varieties (29.85%) than in dryland ones (25.33%) and landraces (20.44%). Among the irrigated varieties, Taimai 101, Xiangmai 23, and Yunhei 161 were most sensitive to water supply, with an increase in GNS by 89.53, 84.23, and 73.57%, respectively. Otherwise, the most insensitive ones were Ziyou 5, Yunyin 1, and Tai 615, and their GNS were increased by 3.01, 3.37, and 3.40%. SN did not change much under two conditions, namely, irrigated varieties and dryland varieties, and landraces were increased by 0.38, 0.95, and 1.24% in I3, respectively. The TGW of irrigated varieties increased the most, 14.30%, that of dryland varieties increased by 6.89%, and that of landrace varieties increased by 3.84%. In the I3 condition, the ASSN decreased by 32.42% in irrigated varieties, more than in dryland (22.93%) and landraces (− 6.46%), whereas the BSSN were less decreased in irrigated varieties (1.26%) than in dryland varieties (10.71%) and landraces (12.17%).

### Correlation Between Traits Under Two Water Regimes

Pearson’s coefficient of correlation between traits was calculated based on the data averaged across 2 years under the two irrigation conditions (Table 2). GNS was significantly positively correlated with SN under both I1 and I3 conditions but was significantly negatively correlated with BSSN under the two irrigation regimes. GNS was also significantly negatively correlated with ASSN under I1. In addition, TGW was slightly positively correlated with GNS but significantly negatively correlated with BSSN under both conditions. Importantly, compared with the I1 treatment, the correlation between GNS and SN, as well as GNS and BSSN, were closer in the I3 treatment, while the correlation between GNS and ASSN was decreased. Results also showed that under both conditions, the correlation coefficients between GNS and both SN and BSSN were highest, while the correlation coefficients between GNS and TGW were lower.

**TABLE 2:** Correlation analysis of different traits for 282 common wheat accessions under the I1 and I3 treatments.

	SN	GNS	BSSN	ASSN	TGW
SN		0.429**	0.194**	0.162**	0.083
GNS	0.302**		−0.509**	–0.022	0.245
BSSN	0.241**	−0.340**		0.091	−0.339**
ASSN	0.181**	−0.263**	0.169**		–0.005
TGW	0.105	0.076	−0.215**	–0.070	

The lower left triangular matrix represents I1; the upper right triangular matrix represents I3. **Indicates significant differences at P < 0.01.

### The Effect of Water Regimes Evaluated by Drought Tolerance Coefficient Value

**FIGURE 1:**
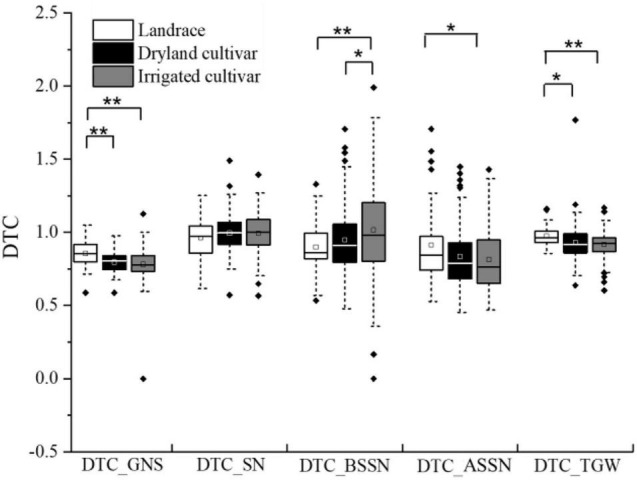
DTC value of related traits in dryland, irrigated cultivars, and landraces. * and ** represent significance level of *P* < 0.05 and *P* < 0.01.

The DTC value for each trait was used to evaluate the influence caused by the different water regimes. DTC_TGW_ and DTC_*SN*_ of most accessions were close to 1 (Figure 1), indicating that these traits of corresponding wheat type were less influenced by water supply. The mean values of DTC_GNS_ in dryland cultivars and irrigated cultivars were 0.81 and 0.79, respectively, and DTC_GNS_ values in landraces were larger than in modern cultivars. The mean value of DTC_BSSN_ and DTC_ASSN_ was less than 1, indicating that the BSSN and ASSN were decreased in the I3 treatment. DTC_ASSN_ was lower than DTC_BSSN_, indicating that compared with BSSN, ASSN was more influenced by water conditions, especially in modern cultivars. Under the two environments, both DTC_ASSN_ and DTC_BSSN_ of this panel showed a larger variation range than DTC_GNS_, DTC_*SN*_, and DTC_TGW_.

### Association Analysis Between Phenotypes and Single Nucleotide Polymorphism Markers

**FIGURE 2:**
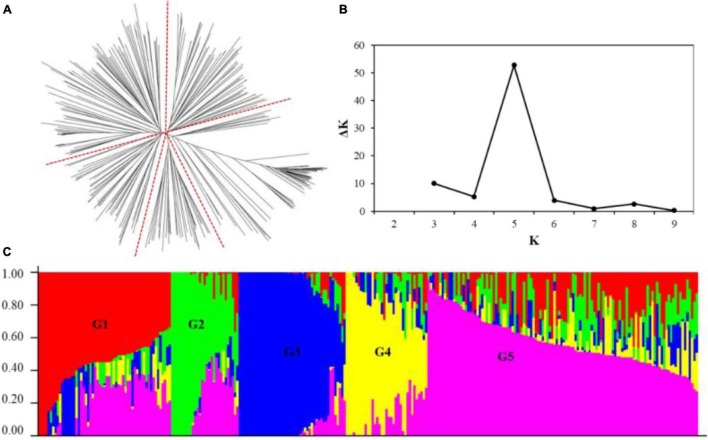
Population structure of the 282 wheat accessions using 9,793 single nucleotide polymorphism (SNP) markers across the whole genome. **(A)** Neighbor-joining tree of the 282 accessions; **(B)** a number of subpopulations estimated by Δ*K* at a range of *K* values; and **(C)** population structure inference of the 282 wheat genotypes based on the SNP marker, using STRUCTURE version 2.3.4.

Two methods were used to analyze the population structure of wheat genotypes from Shanxi. According to the phylogenetic tree constructed using the neighbor-joining method based on Nei’s standard genetic distance, the 282 genotypes were partitioned into five principal groups (Figure 2A). When the number of subpopulations (*K*s) was plotted against the Δ*K* calculated using software STRUCTURE version 2.3.4, the highest Δ*K* was observed at *K* = 5 (Figures 2B,C), which, following the results obtained using the phylogenetic tree, confirmed that the 282 accessions could be divided into five subgroups. The largest group (G5) consists of 115 genotypes, and the other four groups (G1–G4) consist of 57, 30, 45, and 35 genotypes, respectively. Most genotypes belonging to G2 and G4 were modern cultivars developed after 2000. Landraces were grouped into G3. The early-year cultivars, as well as the well-known drought-tolerant wheat accessions such as Jinmai 47 and Chang 6878, were clustered in G1.

The MLM model was used to make association analyses between phenotype traits and SNP markers. GWAS was conducted on four datasets: BLUP-I1, BLUP-I3, BLUP-ALL, and DTC. Significant MTAs [−log_10_ (*p*-value) ≥ 3.0] were identified for the traits in the two water regimes examined. In total, 77 MTAs distributed on 20 chromosomes (except 2D) were identified. Among them, 18, 39, and 20 were on genomes A, B, and D. The phenotypic variation explanation rate (*R*^2^) ranged from 4.62 to 11.19% (Supplementary Table 2). There were 48 and 21 SNPs examined under I1 and I3, respectively. Among them, five SNPs were significantly associated with the same trait under both water regimes. Meanwhile, five SNPs showed significant associations with two or more traits (Supplementary Table 2).

**FIGURE 3:**
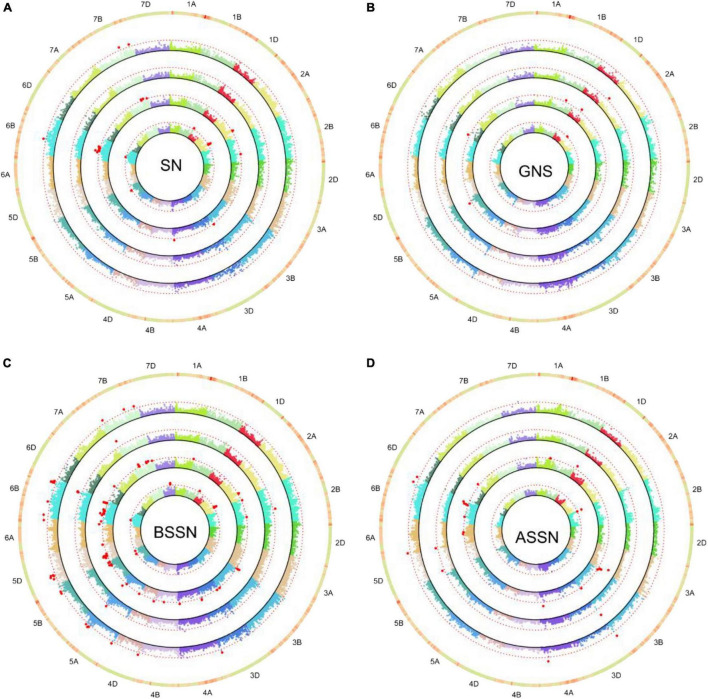
Circular-Manhattan plots for SNP significantly associated with SN **(A)**, grain number per spike (GNS, **B**), the basal sterile spikelet number (BSSN, **C**), and the top sterile spikelet number (ASSN, **D**) under two water regimes identified by genome-wide association study based on the mixed linear model (MLM). Circles from inner to outer represents I1-BLUP, I3-BLUP, BLUP-ALL, and DTC_*trait*_, respectively. The dashed red line represents the threshold –log_10_ (*P*-value) value of 3.0. SNPs markers that met this significant level are highlighted with red dots.

For GNS, nine MTAs were identified on 1B, 1D, 2A, 2B, 3B, 5A, 5B, and 6D. Among these, 4 SNPs under the I1 condition and 4 SNPs under the I3 condition were associated with GNS, respectively. The trait SN was found to be associated with twelve SNPs/genomic regions in I1 and two SNPs in I3. The BSSN trait was associated with the largest number of SNPs: 41 markers/regions were identified in I1 and I3. Twelve markers were found associated with ASSN in the I1 condition and five in the I3 condition. Under both I1 and I3 treatments, four SNPs including *5D_156778694*, *5D_184179300*, *6B_52209942*, and *6B_619721911* were identified for BSSN as well as *3B_806263030* for ASSN (Supplementary Table 2 and Figure 3). *3B_806263030* was associated with not only SN (I1_BLUP and BLUP_ALL) but also ASSN (I1_BLUP, I3_BLUP, and BLUP_ALL). *5A_30786531* was significantly associated with GNS (I3_BLUP) and BSSN (I1_BLUP and BLUP_ALL). *6B_623314284* was significantly associated with ASSN (I1_BLUP and BLUP_ALL) and BSSN (I1_BLUP, I3_BLUP, and BLUP_ALL). *3D_68039763* and *7B_650666608* were significantly associated with both SN and BSSN traits. *5A_575163867* associated with BSSN (575.2 Mb) was colocated with QTLs *qSN5A.3*, *qSL5A.1*, *qGN5A.3*, and *qGS5A.2* (574.6–575.4 Mb) (Pang et al., 2020).

For the DTC values of each trait, eight SNPs/genetic regions were identified in DTC_GNS_ (1), DTC_*SN*_ (4), DTC_BSSN_ (2), and DTC_ASSN_ (1) (Table 3 and Figure 3). Four of them were significantly associated with both trait and its corresponding DTC value, including *2A_82034103* (GNS_I3_BLUP and DTC_GNS_), *2A_567774459* (SN_I1_BLUP and DTC_*SN*_), *2B_3313327* (ASSN_I3_BLUP and DTC_ASSN_), and *6B_283377788* (SN_I1_BLUP, SN_BLUP_ALL, and DTC_*SN*_).

**TABLE 3:** List of significant (*p* < 0.001) marker-DTC_*trait*_ associations detected by GWAS using the MLM model.

Trait	Marker	Chr	Position (Mb)	*F* value	*p*-value	−log_10_ *p*	*R*^2^ (%)
DTC_GNS_	*2A_82034103*	2A	82.03	7.31874	8.05E-04	3.09	4.64
DTC_*SN*_	*1B_668028290*	1B	66.80	7.93578	4.50E-04	3.35	5.83
DTC_*SN*_	*2A_567774459*	2A	547.34-578.37	8.98284	1.68E-04	3.78	6.52
DTC_*SN*_	*5B_448275690*	5B	447.78-448.28	7.61609	6.07E-04	3.22	5.53
DTC_*SN*_	*6B_283377788*	6B	283.38	7.50109	6.77E-04	3.17	5.44
DTC_BSSN_	*5B_603760636*	5B	603.76	7.15395	9.41E-04	3.03	5.32
DTC_BSSN_	*7D_412588658*	7D	408.467-412.59	8.52763	2.57E-04	3.59	6.35
DTC_ASSN_	*2B_3313327*	2B	3.31-5.67	10.30421	4.90E-05	4.31	7.42

### The Distribution of Favored Alleles at Associated Loci

**FIGURE 4:**
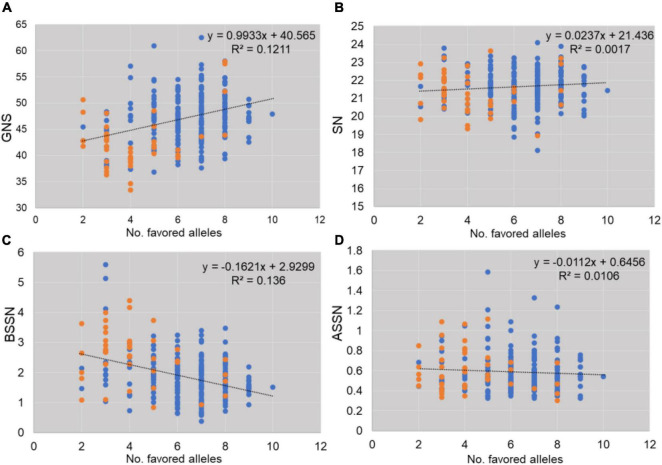
Effects of favorable alleles estimated for traits studied (**A**: GNS, **B**: SN, **C**: BSSN, **D**: ASSN). Blue spots indicate modern varieties and orange spots indicate landraces.

The effect of favored alleles was estimated for GNS investigated in this study (Supplementary Table 3 and Figure 4). Higher allelic effects were found in GNS and BSSN compared with SN and ASSN. Accessions with favorable alleles of locus *2B_26062934_*TT*_*, *6B_52209942_*GG*_*, and *6B_283377788_*GG*_* showed more GNS (increased by 1.09–2.03 with I1 and by 3.29–4.21 with I3; Supplementary Table 3). Genotypes (serial number 74, 89, 150, 151, 180, 259, 269, and 271; Supplementary Table 1) with nine favorable alleles exhibited the considerably higher GNS (40.86–46.76 under I1 and 52.53–70.93 under I3), meanwhile, all these genotypes were found belonging to G5 group in Figure 2C. In addition, the proportion of favored allele for each locus was different, which indicated that these important loci had experienced different degrees of selection during wheat breeding. For example, the proportions of favored alleles for loci *6B_52209942* and *6D_83175038* were 81.91 and 92.20%, respectively, whereas the proportion for locus *2B_132280332* was only 17.73%, which implied that *2B_132280332* had not experienced strong selection. Figure 4 shows that the frequency of favored alleles was much lower in landraces than in modern varieties, which reflected a positive selection of the favorable alleles during the breeding process.

**FIGURE 5:**
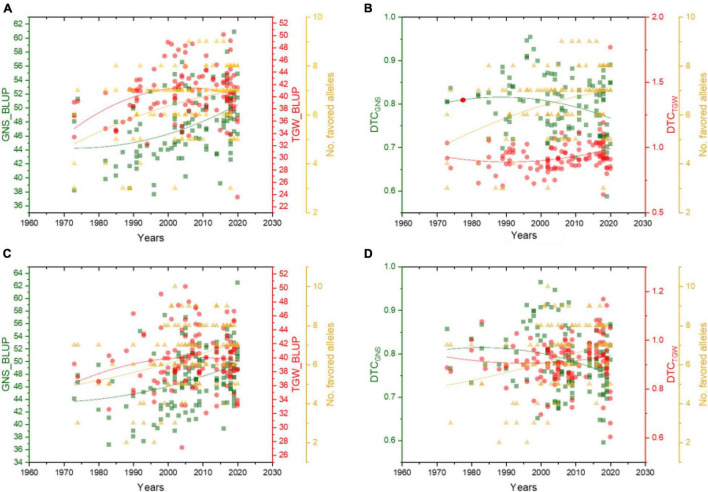
Effect of favorable alleles on GNS, TGW, and their DTC values in dryland **(A,B)** and irrigated wheat **(C,D)** over the years. The red dots represent TGW_BLUP **(A,C)** and DTC_TGW_ **(B,D)**, green squares represent GNS_BLUP **(A,C)** and DTC_GNS_ **(B,D)**, yellow triangles represent the number of favored alleles.

We also analyzed the changing trends of GNS and TGW, as well as the DTC value, of these two traits in dryland and irrigated wheat varieties over the years. Results showed that the GNS of both dryland and irrigated wheat was increased along with the increasing frequency of favored alleles, and the TGW also improved together with GNS over the years (Figures 5A,C). For the dryland varieties, the DTC_TGW_ showed a slight increase over the years; however, the DTC_GNS_ showed a decrease in recent years (Figure 5B), which indicated that the dryland varieties bred in recent years could maintain grain weight if there is not enough water. However, its potential in improving GNS is dependent on sufficient water, which also showed a trend in the present dryland wheat cultivars breeding. Figure 5D shows that, compared with dryland accessions, the irrigated ones exhibited higher DTC_TGW_, indicating that irrigated cultivars were less sensitive in TGW when encountering water deficiency.

## Discussion

A wheat spike normally produces up to 180 floret primordia; however, during development, more than 70% of the florets abort (Guo et al., 2015, 2016). A number of studies showed that the final formation of GNS is determined by the floret initiation and abortion periods (Zhang et al., 2021). Hence, a crucial way to improve grain number is by generating more floret primordia or decreasing floret mortality. The development of young panicles at the jointing stage encounters the differentiation of pistil and stamens to the initial stage of connectivum formation. The water supply at the booting stage is of great significance to the floret development and accumulation of dry matter. This is consistent with the emphasis on irrigation at the jointing and booting stage in wheat production (Cui et al., 2008).

Due to nutritional competition, the increase in spikelet and grain number of a panicle is often accompanied by a decrease in TGW. In this study, there is no significant correlation between SN and TGW or GN and TGW under the I1 and I3 environments (Table 2). Studies have shown that the trade-off between grain number and grain size depends on the growth environment and genotype (Hoang et al., 2019). In the analysis of Shanxi wheat in this study, the GNS and TGW showed a synergistic increase (Figure 5). The GNS and SN showed a low heritability relative to the TGW (Table 1), especially for dryland varieties. The GNS and SN of irrigated wheat showed a strong sensitivity to different water environments. When the water is insufficient, the SN and TGW of dryland wheat do not change so much, while the GNS decreases significantly. This indicates that water affects the firmness of dryland wheat spikelets, thus reducing wheat yield. Therefore, in dryland wheat breeding, we should not blindly select large spikes. For the improvement of fertile spikelets, the yield is increased by increasing the number of grains per spike. The GNS and TGW of irrigated wheat have the lowest response to the water environments and show strong genetic plasticity. Therefore, it is feasible to increase the yield of irrigated wheat by selecting accessions with large spikes and more grains.

Irrigation conditions can also affect the increase in GNS of favored alleles. In Supplementary Table 3, we can find that GNS showed a higher increase in favored alleles under the I3 condition than I1 condition. The impact of the environment on the effect of different haplotypes on grain yield was also observed in the previous reports. For instance, in *SNS_7AL* QTL, the increase in total grain yield in haplotype 2 relative to haplotype 1 was higher in the irrigated treatments than in drought treatments (Kuzay et al., 2019; Voss-Fels et al., 2019). This may be explained by the source-sink changing trends during breeding history. The wheat varieties were changed from weak source and sink strength to considerably weaker source strength and stronger sink. Under the I3 conditions, sufficient water supply promotes the source strength (such as developed leaves) to assimilate enough carbon, which makes the increase in grain yield in cultivars with favored alleles higher (Rodrigues et al., 2019).

As Li et al. (2021) reported in wheat salt tolerance research, two strategies were used to dissect the basis of stress tolerance: one is to identify loci associated with stress tolerance by comparing the different associated markers identified between stress and normal conditions (Hoang et al., 2019; Pradhan et al., 2019; Lin et al., 2020; Ahmed et al., 2021) and the other is to identify associated loci with stress-tolerant indices of investigated traits (Hu et al., 2021; Jeong et al., 2021; Li et al., 2021). In this study, we used these two strategies to identify the GNS-associated markers under water stress. Our results showed that several SNPs for the DTC of related traits were co-localized with SNPs identified under the I1 treatment. For instance, *2A_82034103* for DTC_GNS_, *2A_567774459* for DTC_*SN*_, *2B_3313327* for DTC_ASSN_, these markers were significantly associated with traits under the I1 condition but cannot be examined under the I3 condition (Supplementary Table 2).

## Data Availability Statement

The original contributions presented in the study are included in the article/Supplementary Material, further inquiries can be directed to the corresponding authors.

## Author Contributions

XZ and LQ drafted and revised the manuscript and contributed to data analysis. YL, NW, JJZ, and BW performed the phenotypic evaluation and helped with data analysis. JJZ, BY, and JZ helped to draft the manuscript. JZ and JW designed and coordinated the study and revised the manuscript. All authors have read and approved the final manuscript for publication.

## Conflict of Interest

The authors declare that the research was conducted in the absence of any commercial or financial relationships that could be construed as a potential conflict of interest.

## Publisher’s Note

All claims expressed in this article are solely those of the authors and do not necessarily represent those of their affiliated organizations, or those of the publisher, the editors and the reviewers. Any product that may be evaluated in this article, or claim that may be made by its manufacturer, is not guaranteed or endorsed by the publisher.
