# Supplementary material for: Genome-Wide Association Study of Grain Number in Common Wheat From Shanxi Under Different Water Regimes
Source: Front Plant Sci. 2022 Jan 26;12:806295. doi: 10.3389/fpls.2021.806295 (PMC8825475; doi:10.3389/fpls.2021.806295)
Supplement: Supplementary file 1 [file Table_1.DOCX]

Supplementary Material

## Supplementary Tables

**Supplementary Table 1**. Details information of 282 natural population materials for genome-wide association analysis.

| Number | Accessions | Cultivated ecotype | Year | Number | Accessions | Cultivated ecotype | Year |
| --- | --- | --- | --- | --- | --- | --- | --- |
| 1 | Jinmai1 | dryland wheat | 1973 | 142 | Zhongmai247 | irrigated wheat | 2016 |
| 2 | Jinmai5 | dryland wheat | 1973 | 143 | Linhan6 | dryland wheat | 2006 |
| 3 | Jinmai11 | irrigated wheat | 1980 | 144 | LinY8161 | dryland wheat | 2017 |
| 4 | Jinmai12 | irrigated wheat | 1980 | 145 | Zhonghan110 | dryland wheat | 2002 |
| 5 | Jinmai16 | dryland wheat | 1982 | 146 | Lühan1608 | dryland wheat | 2004 |
| 6 | Jinmai17 | dryland wheat | 1982 | 147 | Xiaoheimai76 | irrigated wheat | 1997 |
| 7 | Jinmai18 | irrigated wheat | 1983 | 148 | Luke298 | irrigated wheat | 2018 |
| 8 | Jinmai19 | irrigated wheat | 1983 | 149 | Jintai141 | dryland wheat | 2017 |
| 9 | Jinmai20 | irrigated wheat | 1984 | 150 | Jintai146 | irrigated wheat | 2017 |
| 10 | Jinmai21 | dryland wheat | 1985 | 151 | Tai1305 | dryland wheat | 2018 |
| 11 | Jinmai22 | dryland wheat | 1985 | 152 | Tai412 | irrigated wheat | 2017 |
| 12 | Jinmai23 | dryland wheat | 1985 | 153 | Yun14guan74 | irrigated wheat | 2018 |
| 13 | Jinmai24 | dryland wheat | 1987 | 154 | Yaomai16 | dryland wheat | 2011 |
| 14 | Jinmai25 | irrigated wheat | 1988 | 155 | Shinong086 | irrigated wheat | 2017 |
| 15 | Jinmai27 | dryland wheat | 1989 | 156 | Xiangmai23 | irrigated wheat | 2017 |
| 16 | Jinmai28 | dryland wheat | 1989 | 157 | Linfen6410 | dryland wheat |  |
| 17 | Jinmai29 | dryland wheat | 1989 | 158 | Jinmai101 | dryland wheat | 2018 |
| 18 | Jinmai31 | dryland wheat | 1990 | 159 | Jinmai102 | dryland wheat | 2018 |
| 19 | Jinmai32 | irrigated wheat | 1990 | 160 | NC206 | irrigated wheat | 2009 |
| 20 | Jinmai30 | irrigated wheat | 1990 | 161 | Zhongmai175 | dryland wheat | 2007 |
| 21 | Jinmai33 | dryland wheat | 1990 | 162 | Shunmai1718 | irrigated wheat | 2011 |
| 22 | Jinmai35 | irrigated wheat | 1990 | 163 | Yunhan1512 | dryland wheat | 2018 |
| 23 | Jinmai36 | dryland wheat | 1991 | 164 | Yunhan139-2 | dryland wheat | 2017 |
| 24 | Jinmai37 | dryland wheat | 1991 | 165 | Runmai2 | dryland wheat | 2016 |
| 25 | Lühan189 | dryland wheat | 1991 | 166 | Xiangmai8156 | dryland wheat | 2017 |
| 26 | Hanxuan11 | dryland wheat | 1991 | 167 | Linhan9 | dryland wheat | 2018 |
| 27 | Hanxuan12 | dryland wheat | 1991 | 168 | Womai323 | dryland wheat | 2018 |
| 28 | Linfen6010 | irrigated wheat | 1992 | 169 | Jinmai919 | dryland wheat | 2018 |
| 29 | Chang86-3768 | dryland wheat | 1992 | 170 | Jinmai104 | dryland wheat | 2017 |
| 30 | Lühan133 | dryland wheat | 1992 | 171 | Chang6789 | irrigated wheat | 2018 |
| 31 | 88-10γ | dryland wheat | 1992 | 172 | LinY7287 | irrigated wheat | 2018 |
| 32 | Linfen7203 | irrigated wheat | 1993 | 173 | Yunhan1411-2 | dryland wheat | 2018 |
| 33 | Taidong1 | dryland wheat | 1994 | 174 | Jinmai9 | irrigated wheat | 1974 |
| 34 | Yun91-15 | dryland wheat | 1995 | 175 | Jinchun14 | irrigated wheat | 1999 |
| 35 | Taiyuan768 | irrigated wheat | 1995 | 176 | Jinchun16 | irrigated wheat | 2009 |
| 36 | Linfen118 | irrigated wheat | 1996 | 177 | Jinchun17 | irrigated wheat | 2017 |
| 37 | Yunfengzao3 | dryland wheat | 1996 | 178 | Yunmai218 | irrigated wheat | 2006 |
| 38 | Jinmai51 | dryland wheat | 1996 | 179 | LinY8012 | irrigated wheat | 2018 |
| 39 | Jinmai52 | irrigated wheat | 1996 | 180 | Womai608 | irrigated wheat | 2018 |
| 40 | Jinmai53 | dryland wheat | 1996 | 181 | Yunhei14207 | irrigated wheat | 2018 |
| 41 | Jinmai54 | dryland wheat | 1997 | 182 | Yunhei161 | irrigated wheat | 2018 |
| 42 | Jinmai56 | irrigated wheat | 1998 | 183 | Yunnuo32 | dryland wheat | 2018 |
| 43 | Jinmai57 | irrigated wheat | 1998 | 184 | Dabaimai | landrace |  |
| 44 | Jinmai58 | irrigated wheat | 1998 | 185 | Xiaohongpi | landrace |  |
| 45 | Jinmai59 | dryland wheat | 1998 | 186 | Niuzhijia1 | landrace |  |
| 46 | Jinmai60 | dryland wheat | 1999 | 187 | Honglimai | landrace |  |
| 47 | Jinmai61 | irrigated wheat | 1999 | 188 | Jiangzhouhong | landrace |  |
| 48 | Jinmai62 | irrigated wheat | 1999 | 189 | Jiahongmai | landrace |  |
| 49 | Jinmai63 | dryland wheat | 1999 | 190 | Niuzhijia2 | landrace |  |
| 50 | Jinmai65 | irrigated wheat | 2000 | 191 | Siyuehuang | landrace |  |
| 51 | Jinmai66 | irrigated wheat | 2000 | 192 | Sanyuehuang | landrace |  |
| 52 | Jinmai67 | irrigated wheat | 2000 | 193 | Baimangcao | landrace |  |
| 53 | Jinmai68 | dryland wheat | 2000 | 194 | Youmangdahongjing | landrace |  |
| 54 | Jinmai70 | dryland wheat | 2001 | 195 | Zhuchengqing | landrace |  |
| 55 | Jinmai71 | irrigated wheat | 2001 | 196 | Hongtumai1 | landrace |  |
| 56 | Jinmai72 | irrigated wheat | 2002 | 197 | Baitumai1 | landrace |  |
| 57 | Jinmai73 | dryland wheat | 2002 | 198 | Baitumai2 | landrace |  |
| 58 | Jinmai74 | irrigated wheat | 2002 | 199 | Hongtumai2 | landrace |  |
| 59 | Jinmai75 | irrigated wheat | 2002 | 200 | Yequmai | landrace |  |
| 60 | Linfeng615 | dryland wheat | 2002 | 201 | Hongpidongmai | landrace |  |
| 61 | Yunyin1 | irrigated wheat | 2002 | 202 | Baishangeda | landrace |  |
| 62 | Jinnong207 | irrigated wheat | 2002 | 203 | Hongyequ | landrace |  |
| 63 | Chang6878 | dryland wheat | 2002 | 204 | Qisifeng | landrace |  |
| 64 | Jintai170 | dryland wheat | 2002 | 205 | Hongheshang | landrace |  |
| 65 | Linyou145 | irrigated wheat | 2003 | 206 | Xianmai | landrace |  |
| 66 | Linfen138 | irrigated wheat | 2003 | 207 | Benmai | landrace |  |
| 67 | Chang6154 | dryland wheat | 2003 | 208 | Baikehong | landrace |  |
| 68 | HedongTX-006 | dryland wheat | 2003 | 209 | Hongxiaomai | landrace |  |
| 69 | Linfeng3 | dryland wheat | 2004 | 210 | Jinguoyin | landrace |  |
| 70 | Jintai65 | irrigated wheat | 2003 | 211 | Youbailan | landrace |  |
| 71 | Linyuan3158 | irrigated wheat | 2004 | 212 | Baixianmai | landrace |  |
| 72 | Linkang11 | dryland wheat | 2004 | 213 | Baigengmai | landrace |  |
| 73 | Zeyou2 | dryland wheat | 2004 | 214 | Baihuomai | landrace |  |
| 74 | Donghei1 | irrigated wheat | 2004 | 215 | Baisanyuehuang | landrace |  |
| 75 | Yunhei28 | irrigated wheat | 2004 | 216 | Yulanmai | landrace |  |
| 76 | Donghei10 | dryland wheat | 2004 | 217 | Zimai | landrace |  |
| 77 | Linyou2018 | irrigated wheat | 2005 | 218 | Datongxiaomai | landrace |  |
| 78 | Linyou2069 | irrigated wheat | 2005 | 219 | Qisuimai | landrace |  |
| 79 | Jinchun3 | dryland wheat | 1974 | 220 | Dahongmai | landrace |  |
| 80 | Jinchun13 | irrigated wheat | 1996 | 221 | Huoshaotou | landrace |  |
| 81 | Jinchun15 | irrigated wheat | 2004 | 222 | Dingxingzhaimai | landrace |  |
| 82 | Yunhan2335 | dryland wheat | 2005 | 223 | Baishanmai | landrace |  |
| 83 | Chang6359 | dryland wheat | 2005 | 224 | Yunhan102 | dryland wheat | 2014 |
| 84 | Changmai5079 | irrigated wheat | 2005 | 225 | Yunhan22-33 | dryland wheat | 2005 |
| 85 | Chang6452 | irrigated wheat | 2005 | 226 | Linhan10 | dryland wheat |  |
| 86 | Chang4640 | dryland wheat | 2005 | 227 | Hanyou6 | dryland wheat |  |
| 87 | Fenheimai1831 | irrigated wheat | 2005 | 228 | Linhan5325 | dryland wheat | 2019 |
| 88 | Jinmai78 | dryland wheat | 2006 | 229 | Chang5804 | dryland wheat |  |
| 89 | Jinmai79 | dryland wheat | 2006 | 230 | Yunhan1818 | dryland wheat |  |
| 90 | Jinmai80 | dryland wheat | 2006 | 231 | Longmai1 | irrigated wheat | 2019 |
| 91 | Jinmai81 | irrigated wheat | 2006 | 232 | Jinmai103 | dryland wheat | 2017 |
| 92 | Fen4846 | irrigated wheat | 2006 | 233 | Yunhan618 | dryland wheat | 2010 |
| 93 | Fen4439 | irrigated wheat | 2006 | 234 | Zhongyou9507 | irrigated wheat | 2001 |
| 94 | Jinmai82 | irrigated wheat | 2007 | 235 | Yunhan115 | dryland wheat | 2011 |
| 95 | Linfen8050 | irrigated wheat | 2007 | 236 | Jintai1510 | dryland wheat | 2018 |
| 96 | Jinmai83 | irrigated wheat | 2007 | 237 | Lumai14 | irrigated wheat | 1993 |
| 97 | Linfen6510 | irrigated wheat | 2007 | 238 | Taimai101 | irrigated wheat | 2018 |
| 98 | Yunhan20410 | dryland wheat | 2007 | 239 | Xinmai296 | irrigated wheat | 2014 |
| 99 | Changmai6686 | irrigated wheat | 2007 | 240 | Xin6160 | irrigated wheat | 2009 |
| 100 | Chang7016 | dryland wheat | 2007 | 241 | Changmai3987 | dryland wheat | 2018 |
| 101 | Jinmai84 | irrigated wheat | 2008 | 242 | Zeyou1 | dryland wheat | 2002 |
| 102 | Jinmai85 | dryland wheat | 2008 | 243 | Shunmai612 | dryland wheat | 2008 |
| 103 | Jintai9923 | irrigated wheat | 2008 | 244 | Zhongyou206 | irrigated wheat | 2008 |
| 104 | Jinmai86 | irrigated wheat | 2008 | 245 | Jinfeng3 | irrigated wheat | 2005 |
| 105 | Tai5902 | irrigated wheat | 2008 | 246 | Changzhi5608 | dryland wheat | 2002 |
| 106 | Changmai6135 | irrigated wheat | 2008 | 247 | Lunxuan167 | irrigated wheat | 2014 |
| 107 | Jinmai87 | dryland wheat | 2009 | 248 | Zhongmai349 | irrigated wheat | 2009 |
| 108 | Yunhan719 | dryland wheat | 2009 | 249 | Chang6388 | dryland wheat | 2019 |
| 109 | Jinmai88 | dryland wheat | 2009 | 250 | Taimai103 | irrigated wheat | 2019 |
| 110 | Shannong129 | irrigated wheat | 2009 | 251 | Jintai1515 | dryland wheat | 2019 |
| 111 | Tai13606 | irrigated wheat | 2009 | 252 | Jinmai106 | irrigated wheat | 2019 |
| 112 | Changmai5973 | irrigated wheat | 2009 | 253 | Jintai1508 | irrigated wheat | 2019 |
| 113 | Chang5222 | irrigated wheat | 2009 | 254 | Chang5638 | irrigated wheat | 2019 |
| 114 | Linyuan8 | irrigated wheat | 2010 | 255 | Jinmai8 | irrigated wheat | 1973 |
| 115 | Changmai251 | irrigated wheat | 2011 | 256 | Changmai6789 | irrigated wheat | 2017 |
| 116 | Chang6197 | dryland wheat | 2017 | 257 | Yun9805 | irrigated wheat | 2002 |
| 117 | Chan8744 | dryland wheat | 2011 | 258 | Zhongmai110 | irrigated wheat | 2019 |
| 118 | Jinmai90 | dryland wheat | 2011 | 259 | Linfen139 | dryland wheat | 2006 |
| 119 | Jinmai91 | dryland wheat | 2011 | 260 | Yongmai3 | irrigated wheat | 2008 |
| 120 | Yunhan805 | dryland wheat | 2011 | 261 | Yunmai766 | irrigated wheat | 2019 |
| 121 | Jinmai92 | dryland wheat | 2013 | 262 | Linxuan2035 | irrigated wheat | 2005 |
| 122 | Jintai182 | irrigated wheat | 2013 | 263 | Linyan151 | irrigated wheat | 2019 |
| 123 | Chang4853 | dryland wheat | 2013 | 264 | Yunmai2064 | irrigated wheat | 2004 |
| 124 | Jinmai94 | irrigated wheat | 2014 | 265 | Shengmai20 | irrigated wheat | 2019 |
| 125 | Jinmai95 | irrigated wheat | 2014 | 266 | Shengmai104 | irrigated wheat | 2019 |
| 126 | Jinmai96 | irrigated wheat | 2014 | 267 | Jinmai107 | dryland wheat | 2019 |
| 127 | Jintai102 | irrigated wheat | 2014 | 268 | Pinyu8155 | dryland wheat | 2019 |
| 128 | Jinmai97 | dryland wheat | 2014 | 269 | Donghei1206 | dryland wheat | 2019 |
| 129 | Jinmai98 | dryland wheat | 2014 | 270 | Taizi6336 | irrigated wheat | 2019 |
| 130 | Taichun3473 | irrigated wheat | 2014 | 271 | Yun85-24 | dryland wheat |  |
| 131 | Jinmai99 | dryland wheat | 2015 | 272 | Jinmai10 | irrigated wheat | 1974 |
| 132 | Yunhan21-30 | dryland wheat | 2003 | 273 | Jinmai89 | irrigated wheat | 2011 |
| 133 | Liangxing67 | irrigated wheat | 2016 | 274 | Jinmai6 | dryland wheat | 1973 |
| 134 | Yunhan137 | dryland wheat | 2016 | 275 | Ziyou5 | irrigated wheat | 2020 |
| 135 | Tai113 | irrigated wheat | 2016 | 276 | Zimai8555 | dryland wheat | 2020 |
| 136 | Jinzuo80 | irrigated wheat | 2016 | 277 | Linmai5311 | irrigated wheat | 2020 |
| 137 | Jintai114 | irrigated wheat | 2016 | 278 | Tai615 | irrigated wheat | 2020 |
| 138 | Chang6794 | irrigated wheat | 2016 | 279 | Changmai3809 | irrigated wheat | 2020 |
| 139 | Chang7080 | dryland wheat | 2017 | 280 | Chang7170 | dryland wheat | 2020 |
| 140 | Jintai1310 | dryland wheat | 2016 | 281 | Linnong4357 | irrigated wheat | 2020 |
| 141 | Chang6990 | dryland wheat | 2016 | 282 | Womai611 | irrigated wheat | 2020 |


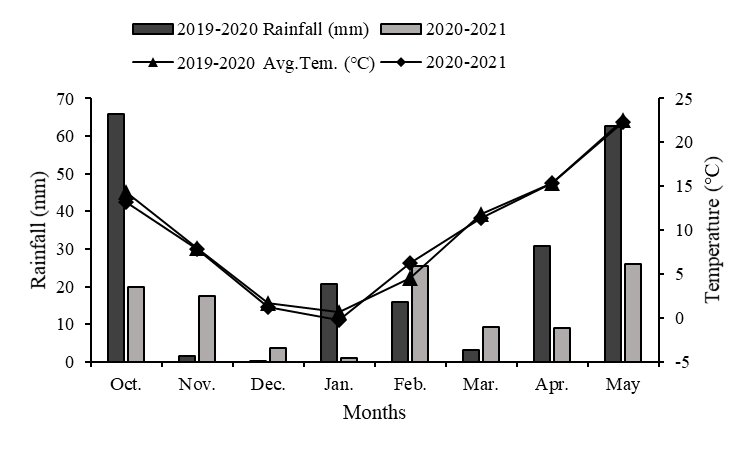


**Supplementary Figure S1**. Monthly rainfall distribution and air temperature during the winter wheat growing seasons in experimental fields at Linfen, Shanxi Province, China in 2019–2020 and 2020–2021.


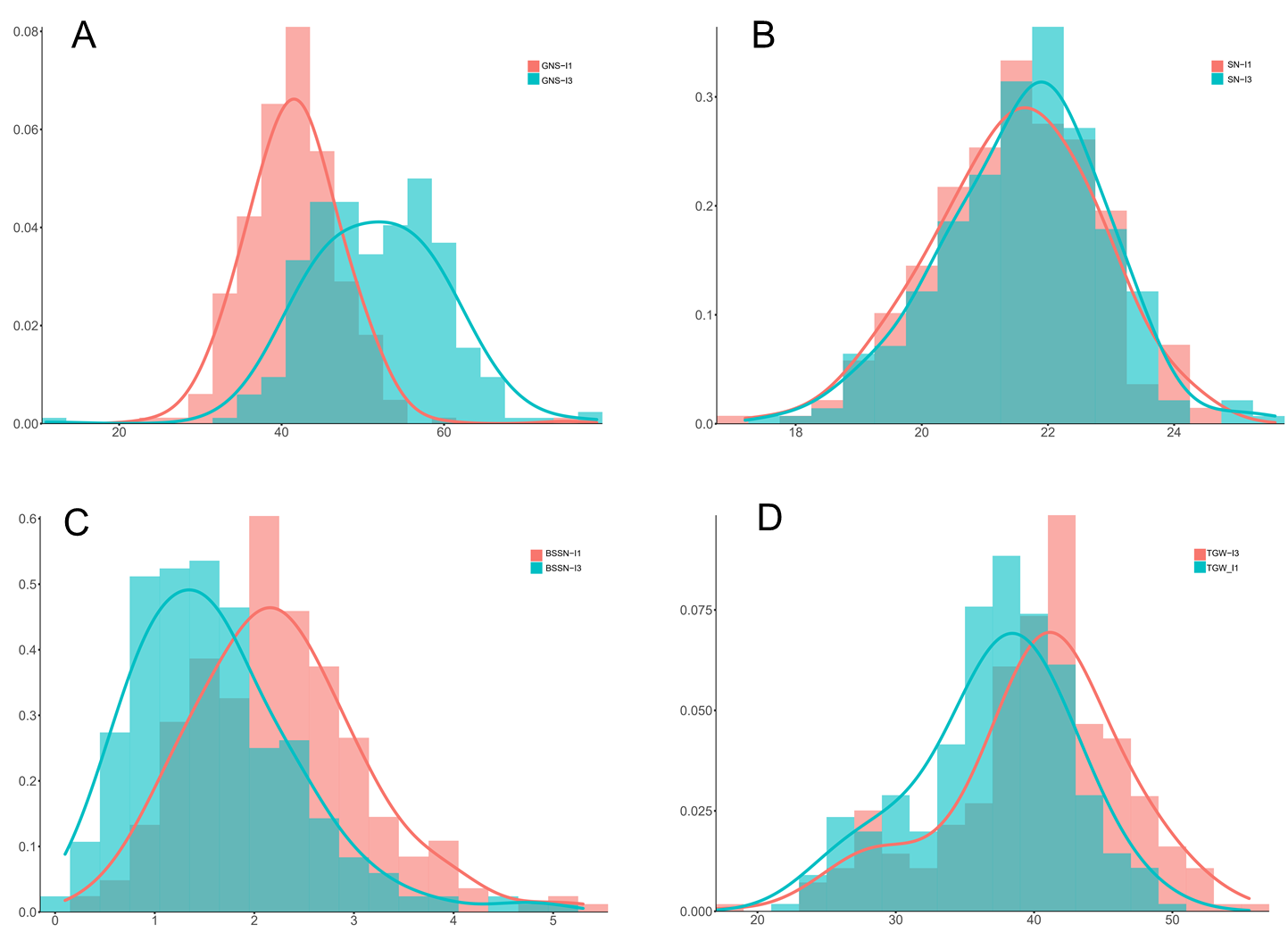


**Supplementary Figure S2**. Frequency distribution of phenotypic variation of the grain number per spike (GNS), total spikelet number per spike (SN), the basal sterile spikelet number (BSSN), thousand grain weight (TGW).

**Supplementary** **Table 2**. List of significant (*p* < 0.001) marker-trait associations detected by GWAS using the MLM model

| **No.** | **Marker** | **Trait** | **Chr** | **Position (bp)** | **F value** | ***p* value** | **LOD** | **R^2^** |
| --- | --- | --- | --- | --- | --- | --- | --- | --- |
| 1 | *1A_436341616* | ASSN_I1_BLUP | 1A | 435582878-436341616 | 7.44512 | 7.13E-04 | 3.15 | 5.494 |
| 2 | *1A_573233513* | BSSN_I1_BLUP | 1A | 573233513 | 7.64883 | 5.90E-04 | 3.23 | 5.494 |
| 3 | *1B_402503625* | GNS_I1_BLUP | 1B | 402503625 | 6.52492 | 2.84E-04 | 3.55 | 7.11 |
| 4 | *1B_589919905* | BSSN_I3_BLUP | 1B | 589919905 | 7.97631 | 4.31E-04 | 3.37 | 5.743 |
| 5 | *1B_648804415* | GNS_I3_BLUP, GNS_BLUP | 1B | 648804415 | 7.33459 | 7.90E-04 | 3.1 | 5.072 |
| 6 | *1B_685724678* | ASSN_I1_BLUP | 1B | 685724678 | 9.41953 | 1.11E-04 | 3.95 | 6.951 |
| 7 | *1D_265344840* | GNS_I1_BLUP | 1D | 265344840 | 6.83733 | 1.91E-04 | 3.72 | 7.79 |
| 8 | *2A_82034103* | GNS_I3_BLUP | 2A | 82034103 | 8.68545 | 2.21E-04 | 3.66 | 6.006 |
| 9 | *2A_567774459* | SN_I1_BLUP | 2A | 567774459-572804008 | 7.84773 | 4.87E-04 | 3.31 | 5.65 |
| 10 | *2B_3313327* | ASSN_I3_BLUP | 2B | 3313327 | 7.97279 | 4.32E-04 | 3.36 | 5.603 |
| 11 | *2B_26062934* | BSSN_I1_BLUP | 2B | 26062934 | 7.71528 | 5.52E-04 | 3.26 | 5.517 |
| 12 | *2B_132280332* | GNS_BLUP | 2B | 132280332 | 7.46891 | 6.95E-04 | 3.16 | 5.284 |
| 13 | *2B_484111600* | BSSN_I3_BLUP | 2B | 484111600 | 8.67718 | 2.22E-04 | 3.65 | 6.248 |
| 14 | *2B_555476388* | SN_I1_BLUP | 2B | 555476388 | 7.29797 | 8.20E-04 | 3.09 | 5.254 |
| 15 | *3A_641899448* | ASSN_I1_BLUP, ASSN_BLUP | 3A | 641899448 | 14.21998 | 1.35E-06 | 5.87 | 10.494 |
| 16 | *3A_732723387* | ASSN_BLUP, ASSN_I1_BLUP | 3A | 732723387 | 9.50991 | 1.02E-04 | 3.99 | 7.018 |
| 17 | *3A_750215533* | BSSN_I1_BLUP | 3A | 747278706-750215533 | 9.26986 | 1.29E-04 | 3.89 | 6.667 |
| 18 | *3B_6931279* | BSSN_I1_BLUP, BSSN_BLUP | 3B | 6931279 | 8.26484 | 3.29E-04 | 3.48 | 5.909 |
| 19 | *3B_40776268* | ASSN_I1_BLUP | 3B | 40776268 | 7.33797 | 7.90E-04 | 3.1 | 5.415 |
| 20 | *3B_44543422* | BSSN_BLUP | 3B | 44543422 | 7.70979 | 5.53E-04 | 3.26 | 5.493 |
| 21 | *3B_81166246* | SN_I3_BLUP | 3B | 81166246 | 8.2495 | 3.32E-04 | 3.48 | 5.79 |
| 22 | *3B_598201289* | GNS_I3_BLUP | 3B | 598201289-636460366 | 7.11186 | 9.76E-04 | 3.01 | 4.918 |
| 23 | *3B_806263030* | SN_I1_BLUP, SN_BLUP, ASSN_I1_BLUP, ASSN_I3_BLUP, ASSN_BLUP | 3B | 793902398-806263030 | 11.75595 | 1.27E-05 | 4.9 | 8.261 |
| 24 | *3D_68039763* | SN_I3_BLUP, SN_BLUP, BSSN_I1_BLUP | 3D | 68039763 | 8.23484 | 3.37E-04 | 3.47 | 5.78 |
| 25 | *3D_222788178* | BSSN_I3_BLUP, BSSN_BLUP | 3D | 222788178 | 8.19738 | 3.50E-04 | 3.46 | 5.903 |
| 26 | *3D_610079744* | BSSN_BLUP | 3D | 610079744-612461886 | 9.55686 | 9.74E-05 | 4.01 | 6.809 |
| 27 | *4A_597902303* | BSSN_I3_BLUP, BSSN_BLUP | 4A | 597902303 | 7.50981 | 6.68E-04 | 3.17 | 5.351 |
| 28 | *4A_610125109* | ASSN_BLUP, ASSN_I1_BLUP | 4A | 610125109 | 9.93226 | 6.86E-05 | 4.16 | 7.05 |
| 29 | *4A_672084473* | SN_I1_BLUP | 4A | 672084473 | 8.1985 | 3.50E-04 | 3.46 | 5.902 |
| 30 | *4A_703248487* | BSSN_BLUP | 4A | 703248487 | 7.3144 | 8.05E-04 | 3.09 | 5.211 |
| 31 | *4B_410665388* | BSSN_BLUP, BSSN_I1_BLUP | 4B | 410665388 | 4.515 | 5.71E-04 | 3.24 | 8.042 |
| 32 | *4D_43461747* | BSSN_BLUP, BSSN_I1_BLUP | 4D | 43461747 | 8.75691 | 2.07E-04 | 3.68 | 6.38 |
| 33 | *5A_30786531* | GNS_I3_BLUP, BSSN_BLUP, BSSN_I1_BLUP | 5A | 27776439-30786531 | 8.61139 | 2.37E-04 | 3.63 | 6.172 |
| 34 | *5A_526290573* | BSSN_BLUP | 5A | 510132281-526650603 | 9.19171 | 1.37E-04 | 3.86 | 6.549 |
| 35 | *5A_575163867* | BSSN_I1_BLUP, BSSN_BLUP | 5A | 575163867 | 9.98944 | 6.73E-05 | 4.17 | 7.683 |
| 36 | *5B_536576057* | GNS_I1_BLUP | 5B | 536576057 | 8.81798 | 1.96E-04 | 3.71 | 6.402 |
| 37 | *5B_546987300* | BSSN_BLUP, BSSN_I1_BLUP | 5B | 546987300 | 8.75042 | 2.07E-04 | 3.68 | 6.235 |
| 38 | *5B_582134236* | BSSN_BLUP, BSSN_I1_BLUP | 5B | 577009211-582134236 | 8.61566 | 2.35E-04 | 3.63 | 6.139 |
| 39 | *5B_662642524* | BSSN_I1_BLUP | 5B | 662642524-664667507 | 8.23747 | 3.37E-04 | 3.47 | 5.89 |
| 40 | *5B_707313542* | SN_BLUP | 5B | 707313542 | 5.95709 | 6.16E-04 | 3.21 | 7.196 |
| 41 | *5D_61214348* | ASSN_I3_BLUP | 5D | 61214348 | 5.62309 | 2.35E-04 | 3.63 | 8.138 |
| 42 | *5D_109938537* | BSSN_BLUP | 5D | 109938537 | 8.05565 | 3.99E-04 | 3.4 | 5.74 |
| 43 | *5D_116251164* | BSSN_BLUP, BSSN_I1_BLUP | 5D | 116251164 | 9.15255 | 1.42E-04 | 3.85 | 6.521 |
| 44 | *5D_133305908* | BSSN_BLUP | 5D | 133305908 | 8.13075 | 3.72E-04 | 3.43 | 5.793 |
| 45 | *5D_150484017* | BSSN_BLUP | 5D | 150484017 | 7.48571 | 6.84E-04 | 3.16 | 5.334 |
| 46 | *5D_156778694* | BSSN_BLUP, BSSN_I1_BLUP, BSSN_I3 | 5D | 156778694 | 10.97272 | 2.61E-05 | 4.58 | 7.818 |
| 47 | *5D_167124713* | BSSN_BLUP, BSSN_I1_BLUP | 5D | 167124713 | 8.40659 | 2.86E-04 | 3.54 | 5.99 |
| 48 | *5D_174462178* | BSSN_I1_BLUP | 5D | 174462178 | 7.88546 | 4.70E-04 | 3.33 | 5.638 |
| 49 | *5D_184179300* | BSSN_BLUP, BSSN_I1, BSSN_I3 | 5D | 184179300 | 10.40498 | 4.42E-05 | 4.35 | 7.414 |
| 50 | *5D_231660382* | BSSN_BLUP, BSSN_I1_BLUP | 5D | 231660382 | 7.67912 | 5.69E-04 | 3.24 | 5.471 |
| 51 | *5D_527496401* | BSSN_I1_BLUP, BSSN_BLUP | 5D | 496136056-527496401 | 12.92571 | 3.85E-04 | 3.41 | 4.621 |
| 52 | *6A_11885037* | ASSN_BLUP, ASSN_I3_BLUP | 6A | 11885037 | 9.03374 | 1.59E-04 | 3.8 | 6.417 |
| 53 | *6A_290475164* | ASSN_I1_BLUP | 6A | 290475164-344534022 | 7.59609 | 6.19E-04 | 3.21 | 5.626 |
| 54 | *6A_579549419* | BSSN_BLUP, BSSN_I1_BLUP | 6A | 579549419 | 9.77851 | 7.99E-05 | 4.1 | 7.075 |
| 55 | *6B_52209942* | BSSN_BLUP, BSSN_I1, BSSN_I3 | 6B | 52209942 | 10.25364 | 5.10E-05 | 4.29 | 7.338 |
| 56 | *6B_195169406* | ASSN_I1_BLUP, ASSN_BLUP | 6B | 195169406 | 7.30894 | 8.12E-04 | 3.09 | 5.394 |
| 57 | *6B_239427241* | SN_I1_BLUP, SN_BLUP | 6B | 239427241 | 8.46258 | 2.73E-04 | 3.56 | 6.092 |
| 58 | *6B_283377788* | SN_I1_BLUP, SN_BLUP | 6B | 283377788-293973178 | 10.02185 | 6.35E-05 | 4.2 | 7.215 |
| 59 | *6B_336758759* | SN_I1_BLUP | 6B | 336758759-340621306 | 7.5401 | 6.52E-04 | 3.19 | 5.54 |
| 60 | *6B_370782094* | SN_I1_BLUP | 6B | 370782094 | 7.78782 | 5.16E-04 | 3.29 | 5.606 |
| 61 | *6B_399603443* | SN_I1_BLUP, SN_BLUP | 6B | 399603443-415774668 | 7.60771 | 6.13E-04 | 3.21 | 5.705 |
| 62 | *6B_491027937* | BSSN_BLUP | 6B | 491027937-492505212 | 8.13419 | 3.72E-04 | 3.43 | 5.87 |
| 63 | *6B_559762733* | ASSN_I1_BLUP, ASSN_BLUP | 6B | 559762733-577829814 | 7.30726 | 8.15E-04 | 3.09 | 5.426 |
| 64 | *6B_623314284* | BSSN_BLUP, BSSN_I1, BSSN_I3, ASSN_I1_BLUP, ASSN_BLUP | 6B | 619720173-633149182 | 6.06386 | 2.46E-05 | 4.61 | 11.187 |
| 65 | *6B_653710307* | BSSN_BLUP | 6B | 653710307 | 8.65141 | 2.28E-04 | 3.64 | 6.164 |
| 66 | *6D_7568338* | ASSN_BLUP | 6D | 7568338 | 8.8623 | 1.87E-04 | 3.73 | 6.291 |
| 67 | *6D_83175038* | GNS_I1_BLUP | 6D | 83175038 | 8.33948 | 3.07E-04 | 3.51 | 6.089 |
| 68 | *6D_460854307* | BSSN_I1_BLUP | 6D | 460854307 | 8.69131 | 2.20E-04 | 3.66 | 6.214 |
| 69 | *7A_84244279* | ASSN_I1_BLUP | 7A | 84244279 | 8.23578 | 3.38E-04 | 3.47 | 6.078 |
| 70 | *7B_66260922* | BSSN_I3_BLUP, BSSN_BLUP | 7B | 66260922 | 11.47667 | 1.64E-05 | 4.78 | 8.264 |
| 71 | *7B_108481257* | BSSN_BLUP | 7B | 108481257 | 7.24409 | 8.60E-04 | 3.07 | 5.161 |
| 72 | *7B_224834472* | BSSN_I1_BLUP, BSSN_BLUP | 7B | 224834472 | 8.83165 | 1.94E-04 | 3.71 | 6.351 |
| 73 | *7B_474404552* | SN_BLUP, SN_I1_BLUP | 7B | 470834142-474404552 | 9.20538 | 1.35E-04 | 3.87 | 6.467 |
| 74 | *7B_538243853* | SN_I1_BLUP | 7B | 538243853 | 7.88985 | 4.68E-04 | 3.33 | 5.68 |
| 75 | *7B_650666608* | SN_BLUP, SN_I1_BLUP, BSSN_I3_BLUP | 7B | 645503356-650666608 | 8.76858 | 2.04E-04 | 3.69 | 6.161 |
| 76 | *7B_713383397* | ASSN_I3_BLUP | 7B | 713383397 | 7.82005 | 4.99E-04 | 3.3 | 5.495 |
| 77 | *7D_326406604* | ASSN_BLUP | 7D | 326406604 | 6.29466 | 3.83E-04 | 3.42 | 6.702 |


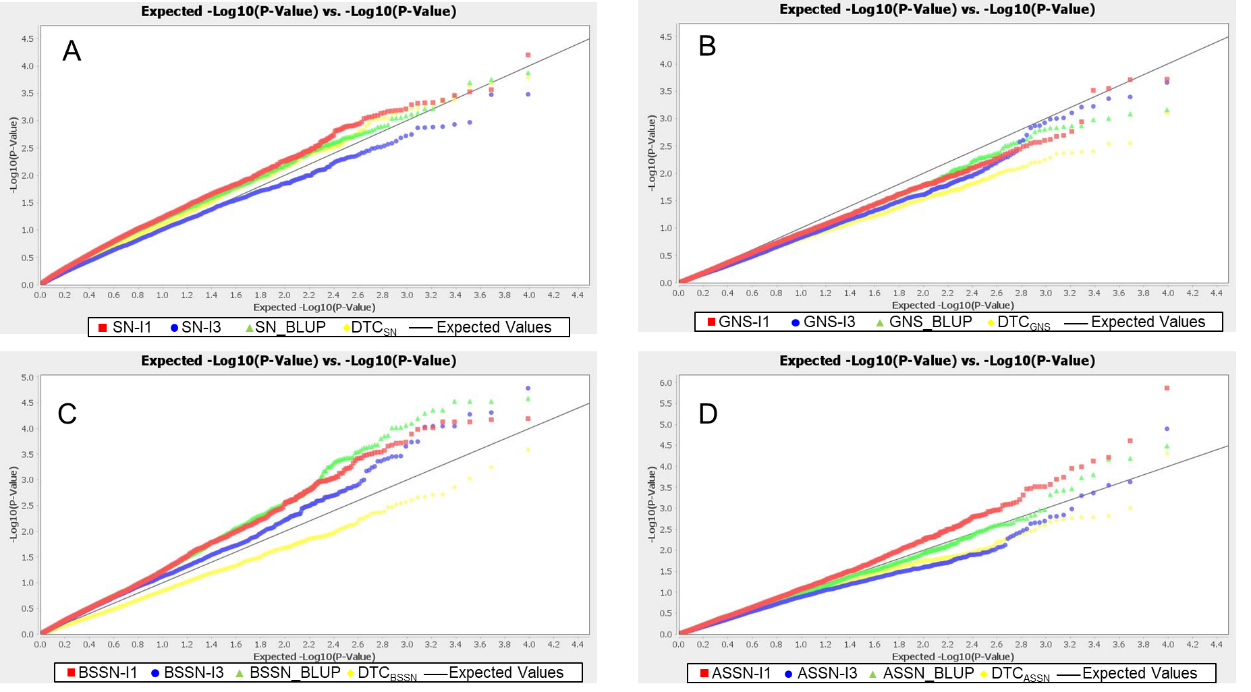


**Supplementary Figure 3.** Quantile–quantile (QQ) plots resulting from the SNP-based GWAS for grain number per spike (GNS) (A), spike number (SN) (B), the basal sterile spikelet number (BSSN) (C), the top sterile spikelet number (ASSN) (D) under two water regimes.

**Supplementary Table 3**. Details information of 282 natural population materials for genome-wide association analysis.

| Marker | Favored allele | | I1 | | | I3 | | | BLUP | | |
| --- | --- | --- | --- | --- | --- | --- | --- | --- | --- | --- | --- |
|  | SNP | Proportion (%) | Favored | Unfavored | Effect | Favored | Unfavored | Effect | Favored | Unfavored | Effect |
| *1B_402503625* | C/T | 25.89 | 41.48 | 40.92 | 0.56 | 53.47 | 51.03 | 2.44 | 47.82 | 45.77 | 2.05 |
| *1B_648804415* | C/T | 62.06 | 41.46 | 40.32 | 1.14 | 53.15 | 48.92 | 4.23 | 47.62 | 43.96 | 3.66 |
| *1D_265344840* | A/G | 70.57 | 41.26 | 40.48 | 0.78 | 52.44 | 50.19 | 2.25 | 46.97 | 44.79 | 2.18 |
| *2B_26062934* | C/T | 69.86 | 41.46 | 40.37 | 1.09 | 52.93 | 49.64 | 3.29 | 47.49 | 44.36 | 3.13 |
| *2B_132280332* | A/G | 17.73 | 42.31 | 40.86 | 1.45 | 54.80 | 51.13 | 3.67 | 49.59 | 45.77 | 3.82 |
| *3B_598201289* | C/T | 23.05 | 41.77 | 40.91 | 0.85 | 54.65 | 51.00 | 3.65 | 48.83 | 45.74 | 3.08 |
| *5A_30786531* | C/T | 32.98 | 41.81 | 40.78 | 1.03 | 54.65 | 50.30 | 4.35 | 48.86 | 45.21 | 3.64 |
| *6B_52209942* | G/A | 81.91 | 41.12 | 39.09 | 2.03 | 51.89 | 47.68 | 4.21 | 46.51 | 41.66 | 4.85 |
| *6B_283377788* | A/G | 37.23 | 41.67 | 40.88 | 0.79 | 52.95 | 51.36 | 1.59 | 47.73 | 45.91 | 1.82 |
| *6D_83175038* | A/G | 92.20 | 41.27 | 40.08 | 1.19 | 52.12 | 50.44 | 1.68 | 46.80 | 44.46 | 2.34 |

Note: red letters indicate the favored alleles.
